# Human genetics uncovers *MAP3K15* as an obesity-independent therapeutic target for diabetes

**DOI:** 10.1126/sciadv.add5430

**Published:** 2022-11-16

**Authors:** Abhishek Nag, Ryan S. Dhindsa, Jonathan Mitchell, Chirag Vasavda, Andrew R. Harper, Dimitrios Vitsios, Andrea Ahnmark, Bilada Bilican, Katja Madeyski-Bengtson, Bader Zarrouki, Anthony W. Zoghbi, Quanli Wang, Katherine R. Smith, Jesus Alegre-Díaz, Pablo Kuri-Morales, Jaime Berumen, Roberto Tapia-Conyer, Jonathan Emberson, Jason M. Torres, Rory Collins, David M. Smith, Benjamin Challis, Dirk S. Paul, Mohammad Bohlooly-Y, Mike Snowden, David Baker, Regina Fritsche-Danielson, Menelas N. Pangalos, Slavé Petrovski

**Affiliations:** 1 Centre for Genomics Research, Discovery Sciences, BioPharmaceuticals R&D, AstraZeneca, Cambridge, UK; 2 Centre for Genomics Research, Discovery Sciences, BioPharmaceuticals R&D, AstraZeneca, Waltham, MA, USA; 3 Bioscience Metabolism, Early CVRM, BioPharmaceuticals R&D, AstraZeneca, Gothenburg, Sweden; 4 Discovery Biology, Discovery Sciences, BioPharmaceuticals R&D, AstraZeneca, Gothenburg, Sweden; 5 Faculty of Medicine, National Autonomous University of Mexico, Copilco Universidad, Coyoacán, 4360 Ciudad de México, Mexico; 6 Nuffield Department of Population Health, University of Oxford, Oxford OX3 7LF, England, UK; 7 Emerging Innovations, Discovery Sciences, BioPharmaceuticals R&D, AstraZeneca, Cambridge, UK; 8 Translational Science and Experimental Medicine, Early CVRM, BioPharmaceuticals R&D, AstraZeneca, Cambridge, UK; 9 Discovery Sciences, BioPharmaceuticals R&D, AstraZeneca, Cambridge, UK; 10 Bioscience Metabolism, Early CVRM, BioPharmaceuticals R&D, AstraZeneca, Cambridge, UK; 11 Early CVRM, BioPharmaceuticals R&D, AstraZeneca, Gothenburg, Sweden; 12 BioPharmaceuticals R&D, AstraZeneca, Cambridge, UK; 13 Department of Medicine, University of Melbourne, Austin Health, Melbourne, Victoria, Australia

## Abstract

We performed collapsing analyses on 454,796 UK Biobank (UKB) exomes to detect gene-level associations with diabetes. Recessive carriers of nonsynonymous variants in *MAP3K15* were 30% less likely to develop diabetes (*P* = 5.7 × 10^−10^) and had lower glycosylated hemoglobin (β = -0.14 SD units, *P* = 1.1 × 10^−24^). These associations were independent of body mass index, suggesting protection against insulin resistance even in the setting of obesity. We replicated these findings in 96,811 Admixed Americans in the Mexico City Prospective Study (*P* < 0.05) Moreover, the protective effect of *MAP3K15* variants was stronger in individuals who did not carry the Latino-enriched *SLC16A11* risk haplotype (*P* = 6.0 × 10^−4^). Separately, we identified a Finnish-enriched *MAP3K15* protein-truncating variant associated with decreased odds of both type 1 and type 2 diabetes (*P* < 0.05) in FinnGen. No adverse phenotypes were associated with protein-truncating *MAP3K15* variants in the UKB, supporting this gene as a therapeutic target for diabetes.

## Introduction

The global burden of diabetes mellitus is projected to grow to 700 million people by 2045, making it one of the fastest growing diseases worldwide (1). It is currently the leading cause of micro- and macrovascular disease, leading to kidney failure, blindness, heart disease, and lower limb amputations (2). Diabetes is broadly categorized into type 1 (T1DM), type 2 (T2DM), and other rarer forms, all of which share the adverse health consequences of persistently elevated blood glucose. T1DM is caused by autoimmune destruction of insulin-producing pancreatic β cells, while T2DM is primarily mediated by peripheral insulin resistance. Both forms of diabetes eventually lead to progressive loss of pancreatic β cells and insufficient insulin secretion (3). Despite the discovery of effective medications for T2DM, such as Glucagon-like Peptide-1 (GLP1) agonists, Dipeptidyl peptidase 4 (DPP4) inhibitors, and sulfonylureas, most of these therapies rely on β cells to secrete insulin. As a result, many patients with T2DM ultimately depend on daily insulin injections once endogenous insulin is no longer available, leaving a substantial unmet need for new targets for therapeutic intervention.

Understanding the genetic contributions to diabetes would help improve our understanding of underlying biological pathways, identify at-risk individuals, and guide more effective precision therapeutics. Genome-wide association studies (GWASs) have identified more than 60 loci for T1DM (4) and hundreds for T2DM (5, 6). With some exceptions, most of these variants map to noncoding regions of the genome, leaving us with few clear candidate genes (5). Without obvious leads, the consequences of these variants on glucose metabolism are challenging to explore mechanistically. GWASs are also limited in scope since they focus on common variants, which tend to have smaller effect sizes.

On the other hand, whole-exome sequencing can uncover the full spectrum of protein-coding variants, including rare and ultra-rare protein-coding variants that have demonstrably large effects on human traits. Of particular interest are loss-of-function alleles that protect against disease since inhibiting their gene products has clear, human-validated precedence for therapeutic intervention (7–9). The growing availability of whole-exome sequences in large populations with linked medical record data has ushered in a new era of gene discovery based on protein-coding variants that could constitute clinically efficacious target opportunities (10).

The largest exome sequencing study for T2DM to date included ~21,000 cases and ~24,000 controls and identified four genes that reached exome-wide significance (11). Here, we report an exome sequencing association study for diabetes in 412,394 multiancestry exomes from the UK Biobank (UKB) with linked health records. This cohort included 33,788 individuals with non–insulin-dependent T2DM, 23,880 with self-reported diabetes, and 4171 with insulin-dependent diabetes. Using our previously developed gene-level collapsing framework (12), we identified that hemizygous protein-truncating variants (PTVs) in the X chromosome gene *MAP3K15* conferred 35% reduced odds of developing diabetes. This protective effect correlated clinically with decreased circulating glucose and hemoglobin A1c (HbAlc) levels. The findings were replicated in two independent cohorts, the Mexico City Prospective Study (MCPS) and FinnGen. Within FinnGen, we identified a particular Finnish-enriched *MAP3K15* PTV that is associated with decreased odds of developing both T1DM and T2DM. PTVs in *MAP3K15* were not associated with any adverse phenotypes in a phenome-wide assessment of 15,719 clinical endpoints in the UKB, suggesting that this gene could be a safe and promising target for managing diabetes.

## Results

### Cohort characteristics and study design

We processed exome sequences from 454,796 UKB participants through our previously described cloud-based pipeline (12). Through stringent quality control, we removed samples with low sequencing quality, with low depth of coverage, and from closely related individuals (Materials and Methods). For this study, we focused on five T1DM- and T2DM-related phenotypes based on self-reported and International Classification of Diseases 10th revision (ICD-10) data: unspecified diabetes mellitus (i.e., self-reported), non–insulin-dependent diabetes mellitus, insulin-dependent diabetes mellitus, “strict” insulin-dependent diabetes mellitus (excluding any individuals who were billed for both non–insulin-dependent and insulin-dependent diabetes), and use of metformin (table S1). In total, 33,788 cases mapped to at least one of the diabetes-related clinical phenotypes. The ancestral breakdown of cases included 30,359 of European ancestry, 2007 of South Asian ancestry, 1234 of African ancestry, and 188 of East Asian ancestry. We also assessed quantitative traits related to diabetes, including nonfasted blood glucose, glycosylated hemoglobin (HbA1c), and body mass index (BMI) (table S2).

We performed single-variant exome-wide association tests (ExWAS) and gene-level collapsing analyses to test for protein-coding associations with each diabetes phenotype (Materials and Methods). As previously described, our collapsing framework tests for genephenotype associations across 18,762 genes under 10 different non-synonymous collapsing models (including a recessive model) to evaluate a range of possible genetic architectures (Materials and Methods and table S3) (12). We performed two versions of the collapsing analysis: one restricted to individuals of European ancestry (~90% of the UKB cohort) and the other a pan-ancestry analysis (Materials and Methods) (12). We did not observe inflation of test statistics in the gene-level collapsing analysis for the five diabetes-related clinical phenotypes tested (median genomic inflation lambda across all models = 1.01).

### European ancestry collapsing analysis and ExWAS

**Fig. 1:**
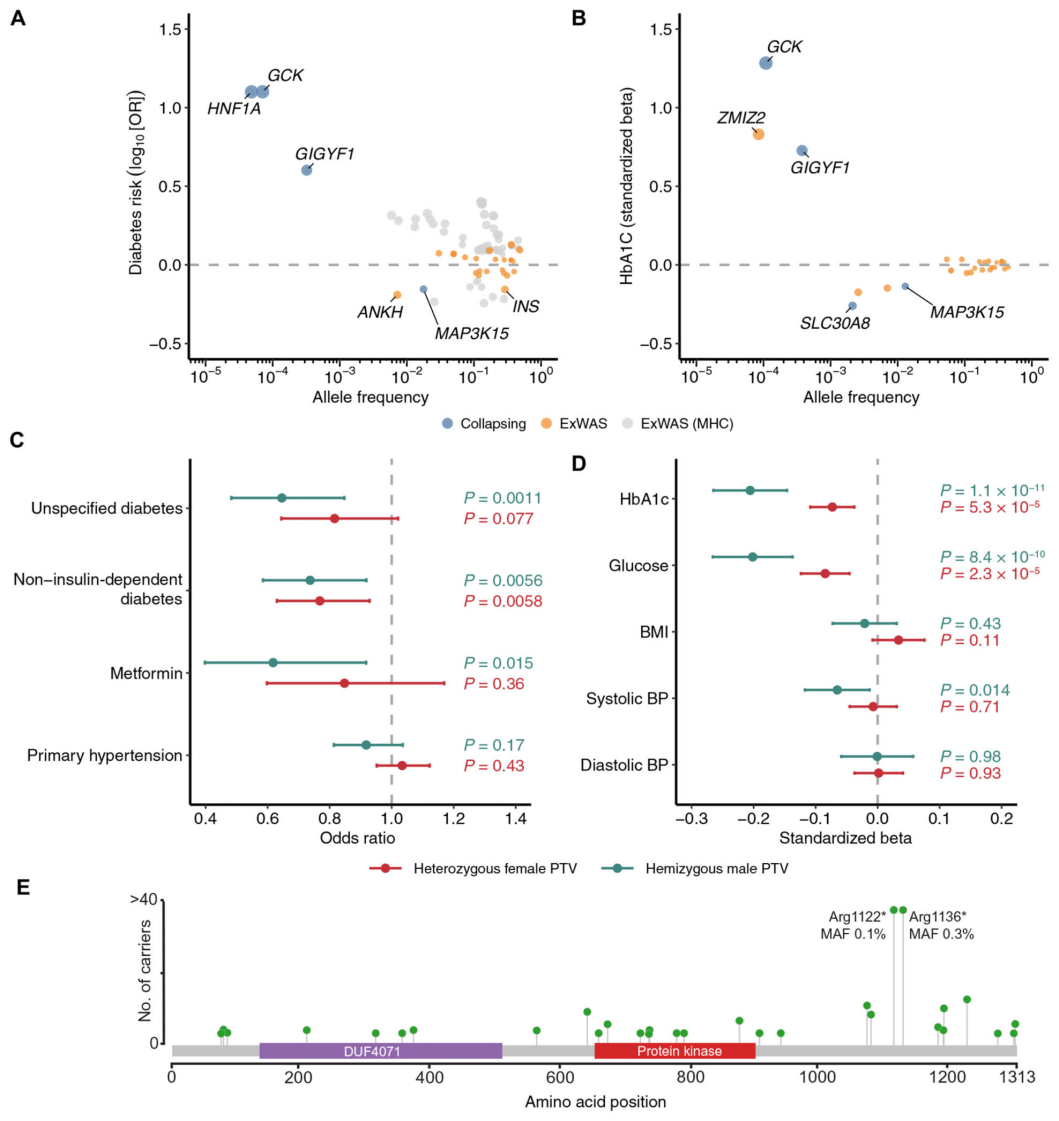
Genetic associations with diabetes and related traits among the European ancestry participants in the UKB. (**A**) ORs and allele frequencies of gene-level collapsing and ExWAS associations (*P* < 1 × 10^−8^) with diabetes diagnoses. (**B**) Effect sizes and allele frequencies of collapsing and ExWAS associations (*P* < 1 × 10^−8^) with HbA1c. We limited associations to those also associated with changes in blood glucose levels. Both (A) and (B) include variants/genes with the largest effect sizes achieved per gene across ExWAS and collapsing models. Allele frequencies for collapsing results are defined as the QV frequency in controls. (**C**) ORs for diabetes and hypertension diagnoses in heterozygous female *MAP3K15* PTV carriers and hemizygous male *MAP3K15* PTV carriers. (**D**) Effect sizes of hemizygous and heterozygous PTVs in *MAP3K15* for various diabetes-related traits. BP, blood pressure. *P* values in (A) and (C) were generated via two-tailed Fisher’s exact test, and *P* values in (B) and (D) were generated via a linear regression model that included age and sex (B) or age (D) as covariates. (**E**) Lollipop plot depicting *MAP3K15* PTVs (stop, gain, and frameshift variants) observed among hemizygous males of European ancestry. Essential splice variants were not included. The *y* axis is capped at 40.

Four protein-coding genes and several individual variants were significantly associated (*P* < 1 × 10^−8^) with at least one diabetes-related clinical phenotype in the European-only collapsing analysis (Fig. 1A, Table 1, and tables S4 to S7). Three genes from the collapsing analysis were associated with increased odds of diabetes and have been reported previously: *GCK, GIGYF1*, and *HNF1A* (13–15). Our recessive collapsing model, which includes homozygous, hemizygous, and putative compound heterozygous carriers of nonsynonymous variants with a minor allele frequency (MAF) < 1%, identified a significant association between *MAP3K15* and self-reported diabetes {odds ratio (OR) = 0.70, 95% confidence interval (CI): [0.62, 0.79], *P* = 5.0 × 10^−9^} (Table 1). Consistent with this, recessive carriers of *MAP3K15* qualifying variants (QVs) had significantly lower HbA1c levels (β = −0.14 SD units, 95% CI: [−0.16, −0.11], *P* = 3.1 × 10 ^23^) (Fig. 1B) and nonfasted blood glucose levels (β = −0.13 SD units, 95% CI: [−0.16, −0.10], *P* = 2.5 × 10^−17^) (table S7). *SLC30A8*, a gene in which loss of function is known to protect against T2DM (16), was the only other gene significantly associated with both reduced HbA1c (“flexdmg” model; β = −0.24 SD units, 95% CI: [−0.30, −0.19], *P* = 1.4 × 10^−17^) and blood glucose (β = −0.19 SD units, 95% CI: [−0.25, −0.13], *P* = 7.3 × 10^−10^) in the collapsing analysis (Fig. 1B).

The association between MAP3K15 and reduced odds of diabetes corroborates our previous findings based on a smaller subset of 269,171 European UKB participants (12). In our prior phenome-wide association study, we found a significant association between recessive nonsynonymous *MAP3K15* variants and reduced HbA1c (β = −0.13 SD units, *P* = 2.16 × 10^−15^) and a suggestive protective association with T2DM (self-reported; OR = 0.73, 95% CI: [0.63–0.85], *P* = 2.71 × 10^−5^). With the increased sample size of 394,692 European participants here, the association between *MAP3K15* and diabetes in the recessive model reached study-wide significance (unspecified/ self-reported diabetes: OR = 0.70; 95% CI: [0.62, 0.79], *P* = 5.0 × 10^−9^), firmly establishing a protective effect of *MAP3K15* loss of function against developing diabetes.

Among the various forms of diabetes, the identified *MAP3K15* variants were most significantly protective against T2DM (non– insulin-dependent diabetes) (table S8). To determine whether recessive variation in *MAP3K15* also protects from T1DM, we defined a T1DM-specific phenotype in the UKB (*N* = 881 cases) using available ICD-10 and primary care information (Materials and Methods). Under the recessive collapsing model, variation in *MAP3K15* appeared to protect against T1DM, but the association did not achieve study-wide significance with the current T1DM sample size (OR = 0.52, 95% CI: [0.25, 1.09], *P* = 0.09) (table S8).

### Heterozygous versus hemizygous *MAP3K15* PTVs

Because the recessive collapsing model includes all nonsynonymous variants, including missense variants, we wanted to test whether the protective mechanism of *MAP3K15* variation operated specifically through recessive loss of function. We thus assessed whether recessive PTVs remained associated with protection from diabetes when missense variants were excluded from the model. Because there were only 5 female homozygous carriers, we focused on hemizygous male (*N* = 1126) and heterozygous female carriers (*N* = 2604) of European ancestry to assess dose-dependent PTV effects.

Heterozygous female carriers had 18% reduced odds of self-reported diabetes compared to female noncarriers (OR = 0.82; 95% CI: [0.64, 1.02], *P* = 0.076) (Fig. 1C and table S9). In comparison, hemizygous male carriers demonstrated a 35% decreased risk of developing diabetes compared to male noncarriers (self-reported; OR = 0.65, 95% CI: [0.48, 0.85], *P* = 0.001; Fig. 1C and table S9). Hemizygous male PTV carriers were also less likely to be prescribed the antidiabetic medication metformin (OR = 0.62, 95% CI: [0.40–0.92], *P* = 0.01) compared to heterozygous females (OR = 0.85, 95% CI: [0.60–1.17], *P* = 0.36) (Fig. 1C). The decrease in HbA1c levels was greater in hemizygous male carriers (β = −0.21 SD units, 95% CI: [−0.26, −0.15], *P* = 1.2 × 10^−11^) (table S10) than in heterozygous female carriers as well (β = −0.07 SD units, 95% CI: [−0.11, −0.04], *P* = 5.3 × 10^−5^) (Fig. 1D). While the decrease in HbA1c appears three times greater in male PTV carriers, the CIs of the point estimates are wide with the current sample size. Future studies with larger sample sizes will increase our confidence in the precise point estimates for hemizygous versus heterozygous PTV carriers. Nonetheless, the effect sizes for diabetes risk, HbA1c, and metformin use in hemizygous *MAP3K15* PTV carriers compared to heterozygous carriers demonstrate that the protective effect of *MAP3K15* loss is dose dependent.

### *MAP3K15* variant-level analyses

We find that PTVs occurred throughout the *MAP3K15* sequence, with two more frequent variants accounting for 74% of the European ancestry hemizygous male carriers: Arg1122* (MAF = 0.11%) and Arg1136* (MAF = 0.35%) (Fig. 1E, fig. S1, and table S11). None of the European ancestry males carried both PTVs despite their proximity. Conditional analysis via logistic regression confirmed that both PTVs were independently associated with reduced odds of diabetes (self-reported) in hemizygous males (Arg1122*: OR = 0.30, 95% CI: [0.12, 0.72], *P* = 0.007; Arg1136*: OR = 0.68, 95% CI: [0.48, 0.97], *P* = 0.035) (table S12). Each variant was also independently associated with lower HbAlc levels in hemizygous males (Arg1122*: β = −0.30 SD units, 95% CI: [−0.44, −0.16], *P* = 4.2 × 10^−5^; Arg1136*: β = −0.19 SD units, 95% CI: [−0.27, −0.11], *P* = 2.0 × 10^−6^) when jointly tested in a linear regression model (table S12). We then performed another gene-level collapsing analysis excluding these two variants and found that the remaining 38 rarer PTVs remained significantly associated with reduced HbA1c in hemizygous males (β = −0.16 SD units, 95% CI: [−0.27, −0.04], *P* = 7.2 × 10^−3^). Hemizygous carriers of the remaining PTVs also appeared to be protected from diabetes (self-reported; OR = 0.83, 95% CI: [0.52, 1.35]), although this association did not achieve statistical significance (*P* = 0.46), likely due to the smaller number of carriers (*N* = 296) (table S12). Last, we ensured that the effect of *MAP3K15* was independent of variation in the nearby *PDHA1* locus (Supplementary Note). Together, these results suggest that loss of function of *MAP3K15* is protective against T2DM.

We next explored the potential mechanisms of recessive *MAP3K15* missense variants. In the ExWAS, 16 recessive missense variants were nominally associated (*P* < 0.05) with HbA1c. Of these, 12 recessive missense variants showed HbA1c-reducing effects (fig. S2). Notably, 6 of these 12 variants had effect sizes at least as strong as the Arg1122* hemizygous PTV (i.e., β ≤ −0.30 SD units). Three of these 12 missense variants had enough allele carriers to be included in the binary trait ExWAS and were associated with reduced odds of diabetes, consistent with their HbA1c-reducing effects (table S13). The remaining four recessive missense variants were nominally associated with increased HbA1c levels (fig. S2). One of these HbA1c-increasing missense variants had enough carriers to be included in the binary trait ExWAS and was associated with increased odds of diabetes (HbA1c: β = 0.10 SD units, *P* = 1.6 × 10^−7^; self-reported diabetes: OR = 1.19, *P* = 0.0062). These results suggest a potential *MAP3K15* allelic series, in which a few missense variants increase disease risk, but PTVs and putatively loss-of-function or hypomorphic missense variants decrease disease risk.

### UKB pan-ancestry analysis

We next tested whether the *MAP3K15* association, as well as the other gene-level diabetes associations, was shared across individuals of African (*n* = 7412), East Asian (*n* = 2209), and South Asian (*n* = 8078) ancestry in the UKB (tables S14 and S15). Under the recessive collapsing model, the ORs for the association between *MAP3K15* and diabetes were consistently in the protective direction for each ancestry (table S15).

We then applied the Cochran-Mantel-Haenszel (CMH) test to combine the results of the full binary trait collapsing analysis results across all four ancestral groups, including Europeans (Materials and Methods). *HNF4A*, which did not reach study-wide significance in the European-only analysis, was significantly associated with increased odds of diabetes in the pan-ancestry analysis (OR = 1.60, 95% CI: [1.37, 1.86], *P* = 5.3 × 10^−9^). Among the genes significantly associated in the European-only collapsing analysis, *GCK* and *GIGYF1* became more significant, while *HNF1A* modestly reduced in significance in the pan-ancestry analysis (table S16). The protective association between *MAP3K15* recessive variants and diabetes (unspecified/self-reported) became more significant in the panancestry analysis (OR = 0.70, 95% CI: [0.62, 0.79], *P* = 5.7 × 10^−10^; table S16). In the pan-ancestry quantitative trait analysis, *MAP3K15* was also more significantly associated with lower HbA1c (β = −0.14 SD units, 95% CI: [−0.16, −0.11], *P* = 1.1 × 10^−24^) and lower nonfasted blood glucose (β = −0.13 SD units, 95% CI: [−0.15, −0.10], *P* = 5.5 × 10^−18^) in the recessive collapsing model (table S17).

### MCPS replication

**Fig. 2:**
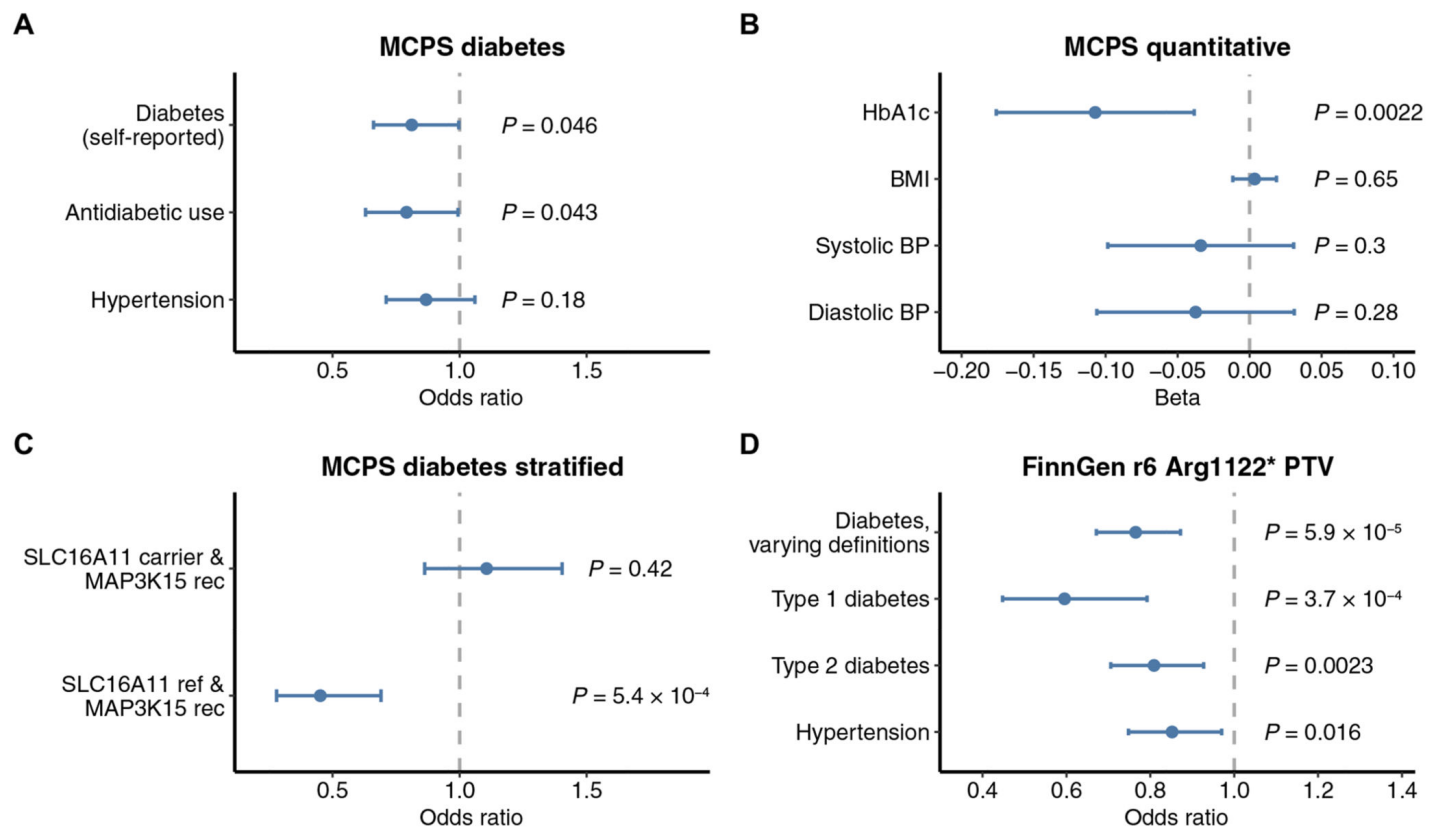
*MAP3K15* replication analyses in MCPS and FinnGen. (**A**) ORs from *MAP3K15* recessive collapsing analysis models for diabetes and hypertension in MCPS. *P* values derived via two-tailed Fisher’s exact test. (**B**) Effect sizes for recessive collapsing analysis of *MAP3K15* and quantitative traits in MCPS. *P* values were generated via linear regression. (**C**) Logistic regression–based stratified analysis of the effect of *MAP3K15* recessive nonsynonymous variants in *SLC16A11* haplotype carriers versus noncarriers (age and sex were included as covariates). *MAP3K15* rec, male or female *MAP3K15* QV carriers under the recessive model. *SLC16A11* ref, carriers of the reference *SLC16A11* haplotype. (**D**) Associations between the Finnish-enriched Arg1122* *MAP3K15* PTV and binary phenotypes in FinnGen (release 6).

In addition to performing pan-ancestry analysis in the UKB cohort, we evaluated the association between *MAP3K15* and diabetes in 96,811 exomes from unrelated individuals of Admixed American ancestry in the MCPS. The prevalence of T2DM in Mexico is among the highest in the world, and in the MCPS, the prevalence of previously diagnosed diabetes rose from 3% at 35 to 39 years of age to greater than 20% by 60 years of age (17). We used the same recessive collapsing model applied in the UKB to test the *MAP3K15* association in this cohort. Recessive nonsynonymous variants in *MAP3K15* were nominally associated with reduced odds of diabetes (self-reported; OR = 0.81, 95% CI: [0.661, 0.996], *P* = 0.046; Fig. 2A and table S18) and were significantly associated with lower HbA1c levels (β = −0.11 SD units, 95% CI: [−0.18, −0.04], *P* = 2.2 × 10^−3^; Fig. 2B and table S19).

Notably, *MAP3K15* PTV carrier frequency (0.38%; *N* = 364 of 96,811) was less than half of that seen in the UKB Europeans (1.1%; *N* = 4191 of 394,692). Consistent with this, European individuals in the Genome Aggregation Database (gnomAD) (18) have the highest frequency of *MAP3K15* PTVs. In contrast, individuals of Mexican or Latin American genetic ancestry have the lowest carrier frequency among all seven represented populations (fig. S3). Thus, populations of European ancestry are most adequately powered for the detection of the protective association between *MAP3K15* and diabetes. To combine evidence for the recessive collapsing model for *MAP3K15* across studies, we extended our original UKB pan-ancestry analysis (comprising of four major ancestral groups) to include the MCPS cohort. In this expanded pan-ancestry analysis, the protective association between recessive nonsynonymous variants in *MAP3K15* and diabetes increased in significance compared to that in the UKB panancestry analysis (CMH OR = 0.73, 95% CI: [0.66, 0.80], *P* = 1.4 × 10^−10^).

The genetic architecture of T2DM is unique in individuals of Mexican descent because a well-known haplotype confers ~20% of this population’s increased risk of disease (19). This haplotype, which contains four missense variants in the gene *SLC16A11*, is exceptionally common in Admixed American individuals (allele frequency ~30%) and rare in Europeans (~1%). Fine-mapping studies and molecular experiments demonstrated that this haplotype results in the lower expression of *SLC16A11*, a transporter that influences fatty acid and lipid metabolism (20). Consistent with prior reports in Mexicans (19), carriers of the *SLC16A11* haplotype in the MCPS cohort were at significantly increased odds of diabetes (OR = 1.37, 95% CI: [1.32, 1.42], *P* = 1.0 × 10^−65^) and had increased HbA1c levels (β = 0.08 SD units, 95% CI: [0.07, 0.09]; *P* = 1.17 × 10^−37^) (Materials and Methods and tables S20 to S22). We thus tested whether variation in *MAP3K15* buffers against the increased disease risk in *SLC16A11* carriers or whether these two genetic factors might operate independently (Materials and Methods). We found a strongly protective effect of recessive nonsynonymous variation in *MAP3K15* in individuals who do not carry the *SLC16A11* risk haplotype (OR = 0.45, 95% CI: [0.28, 0.69], *P* = 5.4 × 10^−4^), which is absent in *SLC16A11* risk haplotype carriers (OR = 1.11, 95% CI: [0.86, 1.40], *P* = 0.42) (table S21). This effect modification was statistically significant under a chi-squared heterogeneity test (χ^2^ = 11.78; 1 df, *P* = 6.0 × 10^−4^). Likewise, HbA1c levels were more strongly reduced in recessive *MAP3K15* carriers who did not carry the *SLC16A11* risk haplotype (β = −0.16 SD units, 95% CI: [−0.26, −0.05], *P* = 0.004) than in those who carried the risk haplotype (β = −0.06 SD units, 95% CI: [−0.15, 0.03], *P* = 0.19) (table S22). These results have important precision medicine implications, suggesting that therapeutically targeting *MAP3K15* may not be as effective in individuals carrying the risk-increasing *SLC16A11* haplotype.

### FinnGen replication analysis

Among ancestral groups in gnomAD (18), PTVs in *MAP3K15* were the second most common in Finnish Europeans (fig. S3). We thus sought to confirm whether the protective association between *MAP3K15* and diabetes was replicated in FinnGen (release 6), which includes genotype data for 260,405 individuals of Finnish descent (21). We found that the Arg1122* PTV (rs140104197) is considerably more enriched in Finnish Europeans than non-Finnish Europeans (MAF: 0.33 versus 0.11%). This enrichment in part reflects the unique advantage of performing genetic analyses in isolated populations, such as Finland, in which alleles that are rare in other populations have increased in frequency due to historical bottlenecks (22). The variant had a high imputation score (INFO score 0.98), reflecting the high confidence in the genotype status of the variant in this dataset. As with the UKB population, we found that individuals carrying the Arg1122* PTV (rs140104197) were significantly protected against T2DM (OR = 0.81, 95% CI: [0.71–0.93], *P* = 2.3 × 10^−3^); additionally, this variant also protected against T1DM in FinnGen (OR = 0.60, 95% CI: [0.45–0.79], *P* = 3.7 × 10^−4^) (table S23). Notably, there are nearly nine times more T1DM cases in FinnGen (*n* = 7609) than in the UKB non-Finnish Europeans (*n* = 881), attributable to Finland having the highest incidence of childhood T1DM globally (23). We were thus better powered to detect the association between *MAP3K15* and T1DM in this population.

### *MAP3K15* protective PTV signal is not associated with changes in BMI or metabolic derangements

Obesity is central to T2DM, both as a risk factor and as a pathologic sequela. Classically, the initial molecular triggers of insulin signaling involve activating the insulin receptor tyrosine kinase and its receptor substrates (24). In obesity, this cascade is disrupted due to the increased activity of several protein phosphatases, which dephosphorylate and terminate signaling (25, 26). As *MAP3K15* encodes a member of the mitogen-activated protein kinase (MAPK) family of signal transducers, we considered whether the protective effects of *MAP3K15* loss of function may be isolated from the upstream consequences of obesity on cell signaling. However, *MAP3K15* appears to be conspicuously specific for glucose metabolism, with little to no effect on other aspects of metabolic syndrome such as blood pressure, BMI, total body fat mass, or body fat percentage in both the UKB and MCPS (Figs. 1, C and D, and 2, C and D, and tables S19 and S24).

To further explore whether the effect of *MAP3K15* on diabetes is independent of obesity, we evaluated whether European individuals with a loss of *MAP3K15* are at a lower risk of developing diabetes even after adjusting for BMI. The protective effects of hemizygous *MAP3K15* PTVs toward both HbA1c (BMI unadjusted: β = −0.21 SD units, 95% CI: [−0.15, −0.26], *P* = 1.2 × 10^−11^; BMI adjusted: β = −0.21 SD units, 95% CI: [−0.15, −0.26], *P* = 4.7 × 10^−12^) and diabetes (BMI unadjusted: OR = 0.65, 95% CI: [0.48, 0.85], *P* = 0.001; BMI adjusted: OR = 0.62, 95% CI: [0.47, 0.83], *P* = 0.001) remained consistent even after adjusting for BMI, demonstrating that the protective effects of losing *MAP3K15* are unlikely to be due to differences in adiposity and are likely to benefit individuals irrespective of BMI.

Some genes that influence diabetes risk can also affect other clinically relevant biomarkers. For example, although PTVs in *GIGYF1* are associated with increased odds of diabetes, they are also associated with reduced low-density lipoprotein (LDL) cholesterol (14). We thus tested for associations between *MAP3K15* and 168 nuclear magnetic resonance (NMR)–based blood metabolite measurements available for 95,077 UKB participants of European ancestry using the recessive collapsing model. Curiously, *MAP3K15* was only associated with reduced glucose (β = −0.11 SD units, 95% CI: [−0.16, −0.06], *P* = 7.2 × 10^−5^) and none of the other metabolites, including those that tend to be deranged in metabolic syndromes, such as triglycerides, LDL cholesterol, and high-density lipoprotein cholesterol (table S25).

### Potential *MAP3K15* safety liabilities

With evidence that loss of *MAP3K15* may be protective against diabetes, targeting *MAP3K15* could become an approach for managing diabetes. However, we first sought to explore whether targeting MAP3K15 may be harmful in humans. Among European participants in the UKB, approximately 1 in every 150 (0.6%) males has a lifetime systemic and complete absence of functional *MAP3K15*. These individuals comprise a generally healthy cohort, suggesting that therapeutically targeting *MAP3K15* function would be tolerable in humans. These patients did not exhibit any worrying changes among the 168 measured blood metabolites. We also evaluated *MAP3K15’s* probability of being loss-of-function intolerant (pLI) score. pLI scores reflect selective pressures against protein-truncating variants (18), with higher scores indicating greater genic intolerance. *MAP3K15’s* pLI score is 0, suggesting that loss of *MAP3K15* is not associated with early-onset phenotypes that affect fecundity.

We also surveyed associations between nonsynonymous variants (12) in *MAP3K15* and 15,719 clinical phenotypes in UKB Europeans. We did not observe any adverse phenotypic associations (*P* < 1 × 10^−4^), including coronary artery or cardiovascular disease, in individuals with loss of *MAP3K15* (“ptv,” “ptv5pcnt,” and “rec” collapsing models) (table S26).

Although a previous animal study reported that loss of *Map3k15* in mice may raise blood pressure (27), we did not find any evidence that individuals harboring *MAP3K15* PTVs were at increased risk of hypertension. In contrast, those with *MAP3K15* PTVs consistently appear less likely to be hypertensive across all three studied global populations: UKB Europeans, FinnGen Finnish Europeans, and Admixed Americans in Mexico City (MCPS). Hemizygous *MAP3K15* PTV status was associated with modestly lower systolic blood pressures (β = −0.07 SD units, 95% CI: [−0.12, −0.01], *P* = 0.01) in UKB Europeans (Fig. 1, C and D, and tables S9 and S10). The Finnish-enriched *MAP3K15* PTV (Arg1122*; rs140104197), which is associated strongly with T1DM and T2DM, was protective against hypertension in the independent FinnGen cohort (OR = 0.85, *P* = 0.016; Fig. 2D and table S23). Last, in the independent MCPS cohort, the same recessive collapsing model that replicated the protective diabetes signal revealed a nonsignificant association with reduced odds of self-reported hypertension (OR = 0.87; 95% CI: [0.71, 1.06], *P* = 0.18; Fig. 2A). The lack of association between recessive variation in *MAP3K15* and any deleterious phenotypes suggests that pharmacologically modulating *MAP3K15* may be safe and worthwhile to explore in humans.

### Supporting evidence

**Fig. 3:**
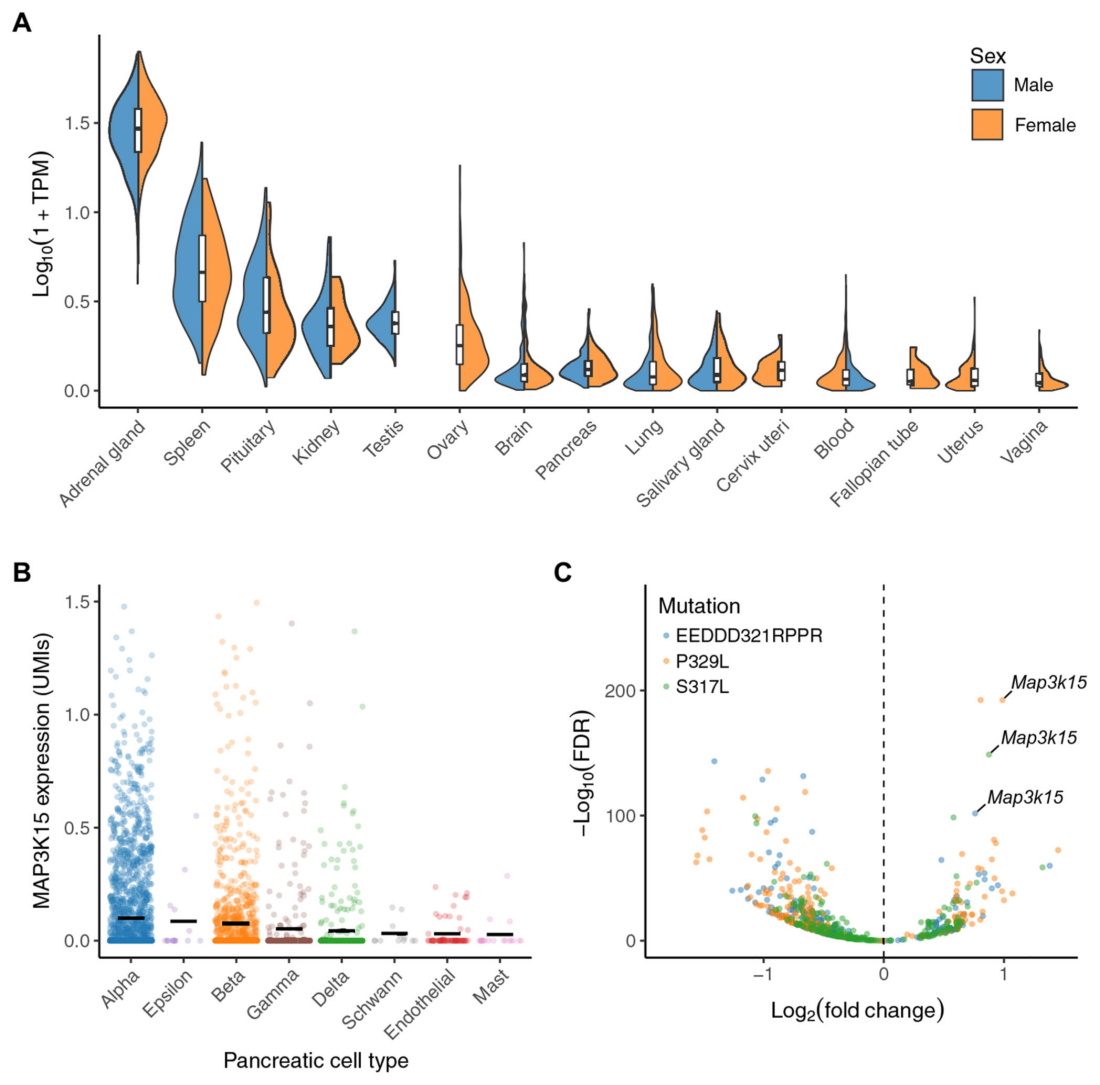
Tissue expression profile of *MAP3K15*. (**A**) Expression of *MAP3K15* in human tissues contained in the GTEx database. TPM, transcripts per million. We only included tissues with a median TPM > 0.1. (**B**) *MAP3K15* expression in major subpopulations of human pancreatic cells derived from a previously published single-cell RNA sequencing dataset (30–34). UMI, unique molecular identifier. (**C**) Volcano plot depicting differential gene expression in mouse insulinoma cell lines stably expressing three variants in *Nkx6-1:* two MODY-associated variants (P329L and S317L) and a control mutation known to functionally impair Nkx6-1 (EEDD321RPPR) (37). FDR, false discovery rate.

Because PTVs in *MAP3K15* appear to reduce the odds of T1DM and T2DM and are not associated with BMI, the protective effect is unlikely to operate through insulin sensitization. Functionally, *MAP3K15* encodes an MAPK known to play major roles in regulating cell stress and apoptotic cell death (28). To gain more insight into how MAP3K15 may influence blood glucose, we examined its expression across tissues within GTEx (29). *MAP3K15* is most strongly expressed in the adrenal glands, but it is also expressed in the spleen, kidney, pancreas, and pituitary glands (Fig. 3A). Single-cell expression data from human pancreatic endocrine cells indicate that *MAP3K15* is most strongly expressed in islet cell subpopulations, including α, β, and δ cells (Fig. 3B) (30–34). Bulk RNA sequencing of pancreatic islet cells from 495 samples contained in the TIGER dataset (35) also revealed increased *MAP3K15* expression in islet cells (fig. S4A). *MAP3K15’s* elevated expression in the pituitary is also intriguing and may reflect some role in growth hormone/insulin-like growth factor 1 signaling. In examining single-cell RNA sequencing data of the developing human adrenal gland (36), *MAP3K15* expression appears to be confined to the adrenal cortex (fig. S4, B and C), suggesting a different potential role in mediating mineralocorticoid or glucocorticoid response.

To further explore how *MAP3K15* may contribute to the pathophysiology of diabetes in pancreatic tissue, we examined its expression in transcriptomic data collected from pancreatic cell lines harboring mutations in *Nkx6-1*, a gene tightly associated with maturity-onset diabetes of the young (MODY) (37). MODY is an early-onset, autosomal dominant presentation of diabetes with a clear heritable component and is thus ripe for studying the genetics of insulin dysregulation and hyperglycemia. One cell line included a mutation known to impair *Nkx6-1* function and served as a positive control, whereas the other two carried MODY-associated variants. Across all three pathologic lines, *MAP3K15* was the most significantly up-regulated gene (Fig. 3C), strongly implicating increased *MAP3K15* activity in the pathophysiology of diabetes. Understanding how *MAP3K15* contributes to MODY will be an important avenue for future work.

**Fig. 4:**
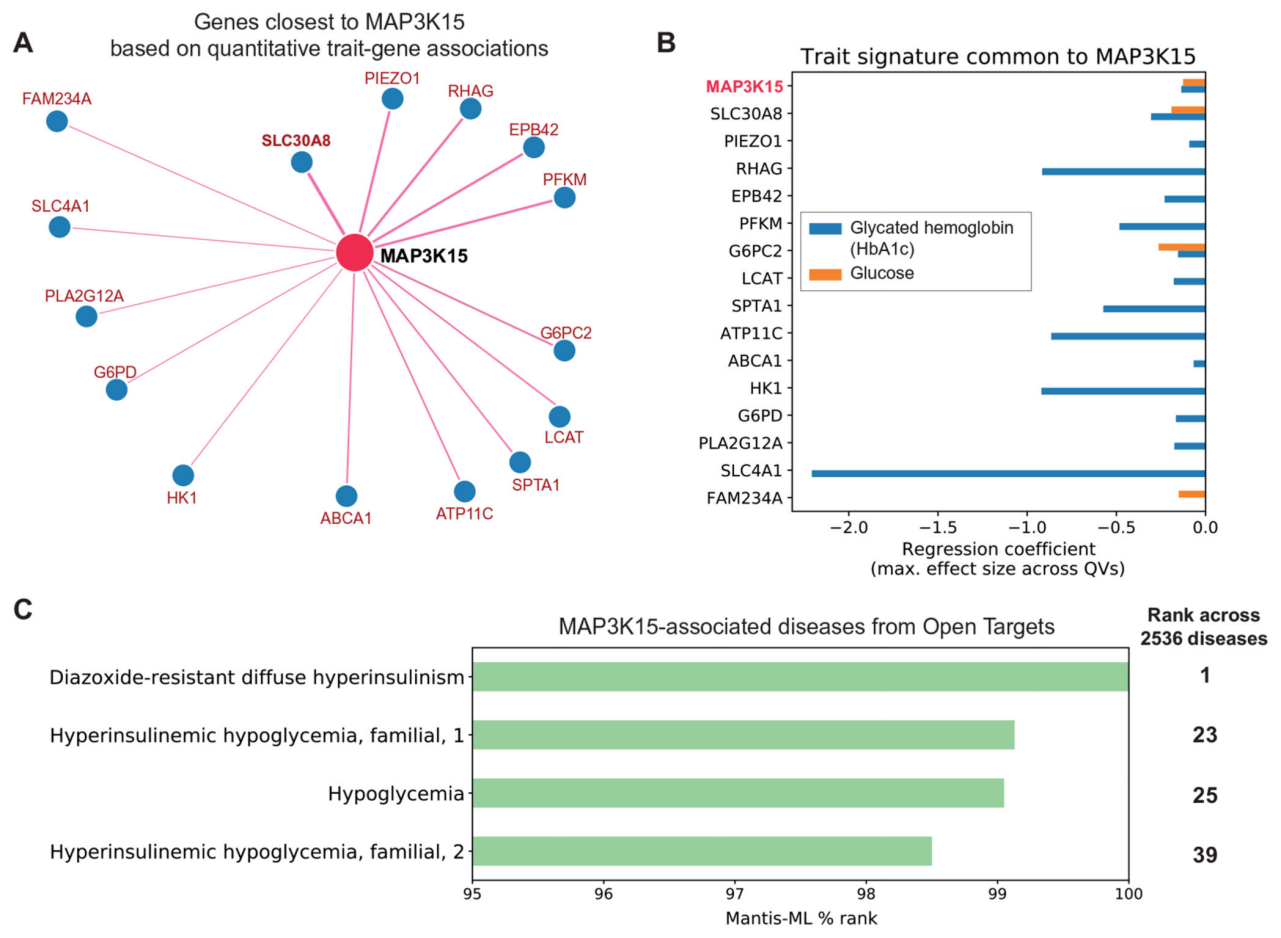
*MAP3K15* quantitative trait and disease signatures. (A) Genes with the most similar quantitative trait profiles to *MAP3K15* in the UKB, derived from Gene-SCOUT (38). (B) Linear regression coefficients for HbA1c and glucose from collapsing analysis models for genes in (A) (genes are sorted from top to bottom in decreasing order of similarity to *MAP3K15*). (C) Mantis ML (41) predictions of *MAP3K15* disease associations.

Evidence derived from two in silico tools further supports the role of *MAP3K15* in diabetes. We first explored Gene-SCOUT, which uses gene-level collapsing analysis statistics for 1419 UKB quantitative traits to identify genes that result in similar biomarker profiles when mutated (38). Using Gene-SCOUT, we found that variation in the zinc transporter gene *SLC30A8* is associated with the most similar human biomarker profile to those with variation in *MAP3K15* (Fig. 4, A and B, and fig. S5). *SLC30A8* is expressed in pancreatic islet α and β cells, with specific variants exerting a protective effect against T2DM, similar to our findings with *MAP3K15* (39, 40).

We also tested whether MAP3K15 was predicted to be associated with diabetes or diabetes-related phenotypes using Mantis-ML (41). This automated machine learning framework predicts potential gene-phenotype relationships using several features, such as tissue expression, genic intolerance, and preclinical models. Among the top 1% of predicted gene-phenotype relationships for *MAP3K15* were “diazoxide-resistant diffuse hyperinsulinism” and “hyper-insulinemic hypoglycemia” (Fig. 4C and table S11). While Mantis-ML does not distinguish between disease-causing and disease-protective effects, these results provide strong evidence that *MAP3K15* is associated with diabetes-related biology.

## Discussion

This exome sequencing study of 456,796 UKB participants increases our understanding of high–effect size genetic factors involved in both propensity for and protection from diabetes in humans. We found that recessive PTVs in *MAP3K15* reduce the odds of developing diabetes by 35% and significantly decrease HbA1c and blood glucose. Although the protective signal was strongest for T2DM, *MAP3K15* PTVs were also protective against T1DM in both the UKB and FinnGen cohorts. Despite being distinct in their etiologies, T1DM and T2DM ultimately share some common pathophysiological pathways such as β cell dysfunction and persistent hyperglycemia (3, 42). Our findings here supply a genetic link between T1DM and T2DM that ties together their shared clinical presentation of hyperglycemia and its many adverse health consequences.

Genes with loss-of-function mutations that protect against human disease present opportune therapeutic targets. As PTVs in *MAP3K15* are strongly associated with lower odds of developing T1DM and T2DM, targeting it may have therapeutic value across the spectrum of diabetes. In our previously published work, *MAP3K15* was 1 of 15 genes strongly associated with glucose and/or HbA1c (12). Another recent independent study on the UKB exomes (43) also suggested a relationship between *MAP3K15* and T2DM among a list of gene-trait associations; however, this observation did not achieve study-wide significance (OR = 0.85, *P* = 2.8 × 10^−6^). In a prior transethnic GWAS, a *MAP3K15* intronic variant was among 318 significant common variant loci reported for T2DM (6). This common variant is not associated with any other complex trait besides T2DM in Open Targets, consistent with our *MAP3K15* PTV-based phenome-wide results (44). With the addition of 150,000 more exomes in the present study, we now observe that loss of *MAP3K15* is associated with a statistically significant reduced risk of diabetes diagnosis in addition to reduced HbA1c. Loss of *MAP3K15* correlates consistently with lower blood glucose and HbA1c levels, which are predictive measures of microvascular sequelae such as peripheral neuropathy, nephropathy, and retinopathy. These convergent associations have important implications for the interpretation of genetic biomarker associations, as genetic associations with clinically relevant biomarkers are not always related to the pathophysiology of a disease. Here, we anchor genetic signals with both biomarkers of diabetes and its clinical diagnosis. Therapeutically, this suggests that targeting *MAP3K15* may influence the pathophysiology underlying diabetes rather than only reducing blood glucose.

While PTVs in *MAP3K15* seem to associate with protection from diabetes broadly, a notable exception was in Admixed American individuals in MCPS who carried the well-known *SLC16A11* risk haplotype (20). Curiously, *SLC16A11* and *MAP3K15* appear to influence different arms of carbohydrate metabolism, and there is no evidence in Search Tool for Retrieval of Interacting Genes/Proteins (STRING) suggesting that these proteins physically interact (45). *SLC16A11* seems particularly important in regulating lipid metabolism by modulating the rates of fatty acid β-oxidation, with knockdown of *SLC16A11* leading to elevated levels of intracellular acylcarnitines and triacylglycerols (45). In contrast, individuals with *MAP3K15* PTVs do not differ much in the serum lipid profile compared to non-PTV carriers but vary significantly in their serum glucose levels. For individuals harboring pathogenic variants in *SLC16A11*, the resulting consequences in lipid metabolism may drive their likelihood of developing diabetes much more so than any effect *MAP3K15* may have on glucose uptake or gluconeogenesis. Regardless, future experimental work would help disentangle these two effects. Because therapeutically targeting *MAP3K15* is likely to be more efficacious in individuals who do not carry the *SLC16A11* risk haplotype, this has potentially important implications regarding precision medicine and clinical trial design.

Through additional phenome-wide association studies in the 454,796 human participants, we find that loss of *MAP3K15* is not associated with any critically adverse phenotypes that would otherwise preclude attempts to target it pharmacologically. Prior work observed that knocking out *Map3k15* in mice led to increased blood pressure (27), but our extensive human study found that *MAP3K15* PTVs appear to provide a protective effect against hypertension.

Although PTVs most often lead to complete loss of protein function, they can also confer partial loss-of-function or, on rarer occasions, even gain-of-function effects. PTVs conferring partial loss- and gain-of-function effects tend to preferentially occur at the 3’ end of a gene and escape nonsense-mediated decay (46). Here, we find that the *MAP3K15* PTV signal is distributed throughout the entire gene body, strongly suggesting a loss-of-function mechanism. Moreover, a prior study found that deletions downstream of amino acid 1179 reduce MAP3K15’s basal kinase activity and render it unable to form molecular condensates in response to osmotic stress (47). Coincidentally, the two more common PTVs that we identified (Arg1122* and Arg1136*) occur upstream of these previously characterized variants. Together, our results suggest that loss of function of MAP3K15 protects against diabetes, but future functional studies will help fully dissect the mechanism of these PTVs.

Exactly how the loss of *MAP3K15* may influence insulin signaling and hyperglycemia is still unclear. The tissue expression profile of *MAP3K15* demonstrates strong expression in several islet cell subpopulations and adrenal glands, suggesting that *MAP3K15* might be involved in pancreatic islet cell functional maintenance and/or stress response pathways. Consistent with this, the *ASK (MAP* kinase) family of genes is known to influence stress response in diabetes (48, 49) (e.g., apoptosis and inflammation) with external stimuli (28). These provide important clues regarding the otherwise unknown pathways that mediate the protective effect between *MAP3K15* and diabetes. Given the notable up-regulation of *MAP3K15* in cellular models of MODY, these models could offer valuable insight into MAP3K15’s role in diabetes.

Although obesity is generally a central driver of type 2 diabetes, we find that the protective effects of *MAP3K15* loss are notably independent of BMI. While not currently available for UKB participants, other quantitative measures of insulin resistance in *MAP3K15* PTV carriers such as fasting glucose, glucose tolerance tests, and a-hydroxybutyrate levels would further illuminate how *MAP3K15* modulates the insulin/glucagon signaling balance and influences hyperglycemia. Nonetheless, our results suggest that pharmacologically targeting *MAP3K15* could be an orthogonal approach to managing diabetes outside the traditional arsenal.

## Materials and Methods

### Discovery cohort

Discovery genetic association studies were performed using the 454,796 exomes available in the UKB cohort (50). The UKB is a prospective study of approximately 500,000 participants aged 40 to 69 years at the time of recruitment. Participants were recruited in the United Kingdom between 2006 and 2010 and are continuously followed. Participant data include health records that are periodically updated by the UKB, self-reported survey information, linkage to death and cancer registries, collection of urine and blood biomarkers, imaging data, accelerometer data, and various other phenotypic endpoints. All study participants provided informed consent, and the UKB has approval from the North-West Multi-centre Research Ethics Committee (11/NW/0382).

### Replication cohorts

#### Mexico City Prospective Study

The MCPS cohort consists of ~150,000 Mexican adults of Admixed American ancestry. Participants were aged at least 35 years and were recruited between 1998 and 2004. Phenotypic data were recorded during household visits. Available phenotypes include age, sex, socioeconomic status, lifestyle factors (e.g., alcohol intake, smoking status, and physical activity), current medications, and medical history (including previously diagnosed diabetes). Height, weight, waist and hip circumferences, and measured blood pressure were measured while the patient was sitting. The full characteristics of this cohort have been described in detail previously (17, 51). The MCPS study was approved by the Mexican Ministry of Health, the Mexican National Council for Science and Technology, and the University of Oxford.

#### FinnGen

The FinnGen cohort (release 6) includes 260,405 individuals from Finland with genotype and health registry data. Phenotypes have been derived from nationwide health registries (21). Patients and control subjects in FinnGen provided informed consent for biobank research, based on the Finnish Biobank Act. Alternatively, older research cohorts, collected before the start of FinnGen (in August 2017), were collected on the basis of study-specific consents and later transferred to the Finnish biobanks after approval by Fimea, the National Supervisory Authority for Welfare and Health. Recruitment protocols followed the biobank protocols approved by Fimea. The Coordinating Ethics Committee of the Hospital District of Helsinki and Uusimaa (HUS) approved the FinnGen study protocol no. HUS/990/2017. The FinnGen study is approved by the Finnish Institute for Health and Welfare.

### Phenotypes

#### UK Biobank

We harmonized the UKB phenotype data as previously described (12). Briefly, we studied two main phenotypic categories: binary and quantitative traits taken from the February 2020 data release that was subsequently refreshed with updated Hospital Episode Statistic and death registry data as released ad hoc by the UKB on July 2020 (UKB application 26041). We parsed phenotypic data using our previously described R package, PEACOCK (https://github.com/astrazeneca-cgr-publications/PEACOK) (12). In addition, as previously described (12), we grouped relevant ICD-10 codes into clinically meaningful “Union” phenotypes. For all binary phenotypes, we matched controls by sex when the percentage of female cases was significantly different (Fisher’s exact two-sided *P* < 0.05) from the percentage of available female controls.

To discover genes associated with the risk of diabetes, we considered five binary diabetes-related phenotypes (table S1): Union#E11#E11 Non-insulin-dependent diabetes mellitus, Union#E14#E14 Unspecified diabetes mellitus, Union#E10#E10 Insulin-dependent diabetes mellitus, Union#E10#E10 Insulin-dependent diabetes mellitus strict (defined as individuals who were never also billed for non–insuli-ndependent diabetes mellitus), and 20003#1140884600#metformin. We also included two related quantitative phenotypes: blood glucose and HbA1c (table S2). When considering HbA1c associations, we specifically focused on genes also associated with changes in blood glucose, as HbA1c can be confounded by any traits that affect red blood cell morphology.

We considered several additional phenotypes in follow-up analyses of *MAP3K15*. In terms of binary phenotypes, we tested for associations with hypertension [Union#I10#I10 Essential (primary) hypertension] and a custom-defined T1DM phenotype that was based on ICD-9 and ICD-10 codes, primary care data, and medication prescriptions. We also analyzed two quantitative traits related to hypertension (systolic blood pressure and diastolic blood pressure) (table S2). In analyzing systolic and diastolic blood pressure, we adjusted for commonly prescribed blood pressure medications in our linear regression collapsing model (described in the “Collapsing analysis” section below) (table S27). Last, we included quantitative traits related to adiposity, including BMI (UKB Field 23104), whole body fat mass (Field 23100), and body fat percentage (Field 23099). All quantitative phenotypes were normalized using rank-based inverse-normal transformation. Effect sizes for these traits are reported as SD units.

#### Mexico City Prospective Study

We assessed three self-reported binary phenotypes in MCPS: recall of a previous diagnosis of diabetes, recall of a previous diagnosis of hypertension, and recall of use of an antidiabetic drug. We also assessed six quantitative traits: baseline HbA1c, diastolic blood pressure adjusted for antihypertensive drug use (plus 10 mmHg), systolic blood pressure adjusted for antihypertensive drug use (plus 15 mmHg), hip circumference, waist circumference, and waist-hip ratio. Collapsing analyses for quantitative traits included BMI as a covariate. Sex matching for each phenotype was performed as described above for the UKB cohort.

#### FinnGen

We extracted all phenotypic associations for one PTV of interest (rs140104197). We focused on four diagnoses: “diabetes (varying definitions),” “type 1 diabetes,” “type 2 diabetes,” and “hypertension, essential.”

### Genetic sequencing

Exome sequencing data for 454,988 UKB participants and 143,440 MCPS participants were generated at the Regeneron Genetics Center. Genomic DNA underwent paired-end 75–base pair whole-exome sequencing at Regeneron Pharmaceuticals using the IDT xGen v1 capture kit on the NovaSeq6000 platform. Conversion of sequencing data in BCL format to FASTQ format and the assignments of paired-end sequence reads to samples were based on 10-base barcodes, using bcl2fastq v2.19.0. Initial quality control was performed by Regeneron and included sex discordance, contamination, unresolved duplicate sequences, and discordance with microarray genotyping data checks. A total of 454,796 UKB exomes and 141,046 MCPS exomes passed these quality control measures.

In FinnGen, genotyping was performed using a Thermo Fisher Scientific Axiom custom array. In addition to the core GWAS markers (about 500,000), it contains 116,402 coding variants enriched in Finland, 10,800 specific markers for the human leukocyte antigen (HLA) and killer cell immunoglobulin-like receptors (KIR) genes, 14,900 ClinVar variants, 4600 pharmacogenomic variants, and 57,000 selected markers.

### AstraZeneca Centre for Genomics Research bioinformatics pipeline

The 454,796 UKB and 141,046 MCPS exome sequences were reprocessed at AstraZeneca from their unaligned FASTQ state. A custom-built Amazon Web Services cloud computing platform running Illumina DRAGEN Bio-IT Platform Germline Pipeline v3.0.7 was used to align the reads to the GRCh38 genome reference and perform single-nucleotide variant (SNV) and insertion and deletion (indel) calling. SNVs and indels were annotated using SnpEFF v4.3 (52) against Ensembl Build 38.92. We further annotated all variants with their gnomAD MAFs (gnomAD v2.1.1 mapped to GRCh38) (18). We also annotated variants using missense tolerance ratio (MTR) scores (53) to identify whether they mapped to genic regions under constraint for missense variants and rare exome variant ensemble learner (REVEL) scores (54) for their predicted deleteriousness.

### Additional quality control

To complement the quality control performed by Regeneron Genomics Centre, we passed the UKB and MCPS exome sequences through our internal bioinformatics pipeline as previously described (12). Briefly, we excluded sequences that achieved a VerifyBAMID freemix (a measure of DNA contamination) of more than 4% and samples where less than 94.5% of the consensus coding sequence (CCDS release 22) achieved a minimum of 10-fold read depth. The cohorts were also screened to remove related participants, as determined using KING v2.2.3 (55): In the UKB, we excluded participants that were second-degree relatives or closer as estimated using the --kinship function (equivalent to kinship coefficient > 0.0884), and in the MCPS, we excluded participants that were first-degree relatives or closer as estimated using the --ibdseg function (equivalent to kinship coefficient > 0.1769). Given the large proportion of related individuals in the MCPS, we followed the following order of prioritizing individuals when doing the relatedness pruning to maximize statistical power for our replication analysis: individuals with a higher number of death records, the presence of a diagnosis of diabetes, a higher number of binary self-reported phenotypes, male predicted sex, and available HbA1c data. After the above quality control steps, there remained 412,394 unrelated UKB and 98,922 MCPS exomes of any genetic ancestry.

### Genetic ancestry

The primary discovery analysis was performed in UKB participants of European ancestry. We predicted the genetic ancestry of UKB participants using PEDDY v0.4.2 (56) with sequences from the 1000 Genomes Project as population references (57). To define the European UKB cohort, we selected individuals with >0.99 Pr(European) ancestry who were within 4 SD of the means for the top four principal components. In total, 394,692 of the 422,488 unrelated UKB exomes (93%) were used for the European ancestry case-control analyses. We also used PEDDY-derived ancestry predictions to identify case-control cohorts from three other major ancestral groups (*N* > 1000) represented in the UKB: African, East Asian, and South Asian. Using a PEDDY cutoff of >0.95 for each of these ancestral groups, we identified 7412 African, 2209 East Asian, and 8078 South Asian UKB participants for case-control analyses.

In MCPS, we retained individuals with PEDDY-derived Pr(Admixed American ancestry) ≥ 0.95. As above, we only retained individuals within 4 SD of the mean for principal components 1 to 4. In total, 96,811 of the 98,922 unrelated MCPS exomes (98%) were of Admixed American ancestry.

### Discovery analyses

#### Collapsing analysis

We performed our previously described gene-level collapsing analysis framework (12) for both binary and quantitative traits. We focused on the European-only analysis as the discovery cohort, given the much larger sample size. We included 10 nonsynonymous collapsing models, including 9 dominant models and 1 recessive model, plus an additional synonymous variant model as an empirical negative control (table S2). For the dominant collapsing models, the carriers of at least one QV in a gene were compared to the noncarriers. In the recessive model, individuals with two copies of QVs in either homozygous or putatively compound heterozygous form were compared to the noncarriers. Hemizygous genotypes for X chromosome genes also qualified for the recessive model.

Using SnpEff annotations, we defined synonymous variants as those annotated as “synonymous_variant.” We defined PTVs as variants annotated as exon_loss_variant, frameshift_variant, start_lost, stop_gained, stop_lost, splice_acceptor_variant, splice_donor_ variant, gene_fusion, bidirectional_gene_fusion, rare_amino_ acid_variant, and transcript_ablation. We defined missense as missense_variant_splice_region_variant and missense_variant. Nonsynonymous variants included exon_loss_variant, frameshift_ variant, start_lost, stop_gained, stop_lost, splice_acceptor_variant, splice_donor_variant, gene_fusion, bidirectional_gene_fusion, rare_amino_acid_variant, transcript_ablation, conservative_inframe_ deletion, conservative_inframe_insertion, disruptive_inframe_insertion, disruptive_inframe_deletion, missense_variant_splice_region_ variant, missense_variant, and protein_altering_variant.

For binary traits, the difference in the proportion of cases and controls carrying QVs in a gene was tested using Fisher’s exact two-sided test. For quantitative traits, the difference in mean between the carriers and noncarriers of QVs was determined by fitting a linear regression model, correcting for age and sex. For analysis of systolic and diastolic blood pressure measurements, we included an indicator variable in the linear regression as a covariate to denote whether individuals were on commonly prescribed antihypertensives (table S27).

For all models, we applied the following quality control filters: minimum coverage 10×; annotation in CCDS transcripts (release 22; approximately 34 Mb); at most, 80% alternate reads in homozygous genotypes; percent of alternate reads in heterozygous variants ≥ 0.25 and ≤ 0.8; binomial test of alternate allele proportion departure from 50% in heterozygous state *P* > 1 × 10^−6^; genotype quality score (GQ) ≥ 20; Fisher’s strand bias score (FS) ≤ 200 (indels) ≤ 60 (SNVs); mapping quality score (MQ) ≥ 40; quality score (QUAL) ≥ 30; read position rank sum score (RPRS) ≥ −2; mapping quality rank sum score (MQRS) ≥ −8; DRAGEN variant status = PASS; the variant site achieved 10-fold coverage in ≥25% of gnomAD exomes; and if the variant was observed in gnomAD exomes, the variant achieved an exome *z* score ≥ −2.0 and exome MQ ≥ 30. We excluded 46 genes that we previously found associated with batch effects (12).

#### Pan-ancestry collapsing analyses

In addition to the European-only analysis described above, we performed the identical collapsing analysis in the South Asian, East Asian, and African UKB cohorts for the five diabetes-related binary traits. We then performed a pan-ancestry analysis, combining the results from these three cohorts and the European cohort using our previously introduced approach (12) of applying a CMH test to generate combined 2 × 2 × *N* stratified *P* values, with *N* representing up to all four genetic ancestry groups. For quantitative traits, we performed a pan-ancestry analysis using a linear regression model that included the following covariates: age, sex, categorical ancestry (European, African, East Asian, or South Asian), and top five ancestry principal components.

#### European ancestry ExWAS

We performed variant-level association tests in addition to the gene-level collapsing analyses for the five binary and five quantitative traits related to diabetes. We tested 3.3 million variants identified in at least six individuals from the 394,692 predominantly unrelated European ancestry UKB exomes as previously described (12). In summary, variants were required to pass the following quality control criteria: minimum coverage 10×; percent of alternate reads in heterozygous variants ≥ 0.2; binomial test of alternate allele proportion departure from 50% in heterozygous state *P* > 1 × 10^−6^; GQ ≥ 20; FS ≤ 200 (indels) ≤ 60 (SNVs); MQ ≥ 40; QUAL ≥ 30; RPRS ≥ −2; MQRS ≥ −8; DRAGEN variant status = PASS; the variant site is not missing (that is, less than 10× coverage) in 10% or more of sequences; the variant did not fail any of the aforementioned quality control in 5% or more of sequences; the variant site achieved 10-fold coverage in 30% or more of gnomAD exomes; and if the variant was observed in gnomAD exomes, 50% or more of the time, those variant calls passed the gnomAD quality control filters (gnomAD exome AC/AC_raw ≥ 50%). *P* values were generated by adopting Fisher’s exact two-sided test. Three distinct genetic models were studied for binary traits: allelic (A versus B allele), dominant (AA + AB versus BB), and recessive (AA versus AB + BB), where A denotes the alternative allele and B denotes the reference allele. For quantitative traits, we adopted a linear regression (correcting for age and sex) and replaced the allelic model with a genotypic (AA versus AB versus BB) test.

#### Phenome-wide analysis for MAP3K15

We performed phenome-wide associations between *MAP3K15* and 15,710 binary phenotypes and 1419 quantitative phenotypes in the 394,692 European UKB individuals using the identical parameters published in our prior PheWAS publication (12). We have made all statistics publicly available through our PheWAS portal (https://azphewas.com/geneView/7e2a7fab-97f0-45f7-9297-f976f7e667c8/MAP3K15/glr/binary).

#### P value threshold

We defined the study-wide significance threshold as *P* < 1 × 10^−8^. We have previously shown, using an *n*-of-1 permutation approach and the empirical null synonymous model, that this threshold corresponds to a false-positive rate of 9 (of a total of 3.6 billion tests) and 2 (of a total of 346.5 million tests), respectively, for binary traits in the setting of collapsing analysis PheWAS (12).

### Secondary association analyses

A total of 40 unique PTVs in *MAP3K15* were observed among the hemizygous male carriers. Two of these PTVs (Arg1122* and Arg1136*) were relatively more frequent. We excluded carriers of these two alleles and reperformed the collapsing analyses for the remaining *MAP3K15* PTVs: Fisher’s exact test for diabetes (“Union#E14#E14 Unspecified diabetes mellitus”) and linear regression for HbA1c.

To determine whether the protective effect of *MAP3K15* on diabetes is independent of BMI, we performed additional analyses in which we regressed HbA1c and the self-reported diabetes phenotype (Union#E14#E14 Unspecified diabetes mellitus) on *MAP3K15* PTV carrier status in males with BMI (UKB Field ID: 23104) as a covariate. To investigate the joint effects of *MAP3K15* and a nearby significantly associated indel in *PDHA1* (X-19360844-AAC-A), a gene that overlaps the 3’ untranslated region of *MAP3K15*, we regressed HbA1c and the diabetes phenotype (Union#E14#E14 Unspecified diabetes mellitus) on the carrier status for the two frequent *MAP3K15* PTVs (Arg1122* and Arg1136*) and the *PDHA1* indel in hemizygous males.

### MCPS *SLC16A11* analysis

A common haplotype spanning the *SLC16A11* gene that harbors four missense variants (17-7041768-G-T, 17-7042164-C-T, 17-7042968-T-C, and 17-7043011-C-T) has been previously associated with T2DM risk in Latin American populations (19). We tested whether each of these four *SLC16A11* missense variants was associated with self-reported diabetes in the MCPS cohort, using a dominant logistic regression model with age and sex as covariates. Each missense variant showed roughly the same level of association with diabetes (table S20). Moreover, we estimated the extent of linkage disequilibrium (LD) between the missense variants in the MCPS cohort [using the --ld function on PLINK v2.0 (58)], which showed that they were in strong LD with one another (all pairwise *D’* = 1 and *r*^2^ > 0.7). We selected the variant 17-7041768-G-T to test this risk haplotype for downstream analyses.

We performed a stratified analysis in which we tested the association between recessive carriers of *MAP3K15* variants and self-reported diabetes in carriers and noncarriers of the 17-7041768-G-T variant. Recessive *MAP3K15* carriers were those who met the QV criteria for the recessive collapsing models. We performed the association test using a logistic regression model correcting for age and sex as covariates. For the HbA1c stratified analysis, we used linear regression in place of logistic regression, also correcting for age and sex.

### Metabolomics

As detailed in a prior publication (59), 168 blood metabolites, including lipoprotein lipids, fatty acids and their compositions, and various low–molecular weight metabolites, were profiled in a subset of ~ 120,000 UKB participants by Nightingale Health using NMR spectroscopy. We performed association analyses on the subset of these individuals who were of European ancestry (*N* = 95,077). We used the same quality control and normalization procedure published in our recent publication on UKB biomarkers (60). Briefly, we applied a rank-based inverse-normal transformation to the measurements and corrected several cholesterol measurements for commonly prescribed medications. We then performed a quantitative collapsing analysis limited to *MAP3K15* using the recessive model.

### Expression analyses

We studied previously published bulk RNA sequencing data available from a mouse insulinoma cell line (β-TC-6) transfected with three different clones carrying MODY-associated variants in NKX6-124. We extracted the DESeq2-derived log fold changes, *P* values, and false discovery rate values from the supplementary data. We determined tissue expression using the GTEx portal (http://gtexportal.org/home/). For single-cell pancreas RNA sequencing analysis, we examined eight previously published datasets using tissue from human pancreatic islets spanning 27 healthy donors, five technologies, and four laboratories (30–34). Preprocessed and annotated data were downloaded using the SeuratData package and then integrated using Seurat, as previously described (61). We retrieved previously published data on the developing human adrenal cortex from eight human samples (36). The preprocessed Seurat object with annotated cell types was downloaded from https://github.com/artem-artemov/adrenal.

### Gene-SCOUT

The tool Gene-SCOUT (38) estimates similarity between genes by leveraging association statistics from the collapsing analysis across 1419 quantitative traits available in the UKB. We used this tool to identify genes that were most similar to the “seed gene” *MAP3K15*.

### Mantis-ML

Mantis-ML (41) is a gene prioritization machine learning framework that integrates a diverse set of annotations, including intolerance to variation, tissue expression, and animal models. We used this tool to obtain the top disease predictions for *MAP3K15* across 2536 diseases parsed from Open Targets.

## Supplementary Material

Figs. S1 to S5

Tables S1 to S27

## Figures and Tables

**Table 1:** Significant gene-level collapsing associations with diabetes in European UKB participants. Association statistics for the four genes that were significantly associated with at least one diabetes phenotype (*P* < 1 × 10^−8^). A complete list of associations is provided in table S4. Collapsing models are defined in table S3.

Gene	Phenotype	Case freq.	Ctrl. freq.	OR [95% CI]	*P*	Model
***GCK***	Union#E11#E11 Non–insulindependent diabetes mellitus	0.21%	0.04%	5.25 [3.84–7.17]	8.2 × 10^−21^	URmtr
***GIGYF1***	Union#E11#E11 Non–insulindependent diabetes mellitus	0.13%	0.03%	4.00 [2.74–5.84]	1.0 × 10^−10^	ptv
***HNF1A***	Union#E14#E14 Unspecified diabetes mellitus	0.06%	0.005%	12.61 [5.85–27.21]	7.6 × 10^−9^	ptv5pcnt
***MAP3K15***	Union#E14#E14 Unspecified diabetes mellitus	1.25%	1.77%	0.70 [0.62–0.79]	5.0 × 10^−9^	rec

## Data Availability

UKB association statistics generated in this study are available both in the Supplementary Materials and through our AstraZeneca Centre for Genomics Research (CGR) PheWAS Portal (http://azphewas.com/). All UKB whole-exome sequencing data described here are publicly available to registered researchers through the UKB data access protocol. Exomes can be found in the UKB showcase portal: https://biobank.ndph.ox.ac.uk/. Additional information about registration for access to the data is available at www.ukbiobank.ac.uk/register-apply/. Data for this study were obtained under Resource Application Number 26041. The Mexico City Prospective Study welcomes open access and collaboration data requests. Researchers interested in accessing such data should visit the study website (www.ctsu.ox.ac.uk/research/prospective-blood-based-study-of-150-000-individuals-in-mexico) where the MCPS Data and Sample Sharing Policy can be downloaded in either English or Spanish. FinnGen release r6 association statistics are publicly available (http://r6.finngen.fi). GTEx bulk RNA sequencing data are available at http://gtexportal.org/home/. Pancreas single-cell RNA sequencing data (“panc8”) are available at https://github.com/satijalab/seurat-data, and adrenal cortex single-cell RNA sequencing is available at https://github.com/artem-artemov/ adrenal. PheWAS and ExWAS association tests were performed using a custom framework, PEACOK (PEACOK 1.0.7). PEACOK 1.0.7 is available on Zenodo (https://doi.org/10.5281/zenodo.7097303) and GitHub (https://github.com/astrazeneca-cgr-publications/PEACOK/). For the purpose of open access, the authors have applied a Creative Commons Attribution (CC BY) license to any Author Accepted Manuscript version arising.
