# Supplementary material for: Human genetics uncovers MAP3K15 as an obesity-independent therapeutic target for diabetes
Source: Sci Adv. Author manuscript; Available in PMC 2022 Dec 5. (PMC9668288; doi:10.1126/sciadv.add5430)
Supplement: Figs. S1 to S5 [file EMS157313-supplement-Figs__S1_to_S5.pdf]

Science Advances
AAAS

Supplementary Materials for

Human genetics uncovers *MAP3K15* as an obesity-independent therapeutic target for diabetes

Abhishek Nag *et al.*

Corresponding author: Ryan S. Dhindsa, ryan.dhindsa@astrazeneca.com;
Slavé Petrovski, slav.petrovski@astrazeneca.com

Sci. Adv. **8**, eadd5430 (2022)
DOI: 10.1126/sciadv.add5430

The PDF file includes:

Supplementary Note
Figs. S1 to S5
Legends for tables S1 to S27

Other Supplementary Material for this manuscript includes the following:

Tables S1 to S27

Supplementary Note

<u>Joint analysis of the *MAP3K15* and *PDHA1* loci</u>

MAP3K15 overlaps with the 3'-UTR of *PDHA1,* a gene that encodes a subunit of the enzyme pyruvate dehydrogenase and catalyzes a step in the glycolysis pathway. Moreover, an indel (X-19360844-AAC-A) in the 3'-UTR of *PDHA1* is significantly associated with HbA1c levels in the UKB (beta = -0.13, 95% CI: [-0.17, -0.09], $P = 2.1 \times 10^{-11}$). We performed a conditional analysis to ensure that the observed effect of the two more common *MAP3K15* PTVs on HbA1C levels was independent of the *PDHA1* indel. Indeed, the associations for all three variants with HbA1c remained significant in the joint analysis (**Supplementary Table 12**).

Figure S1

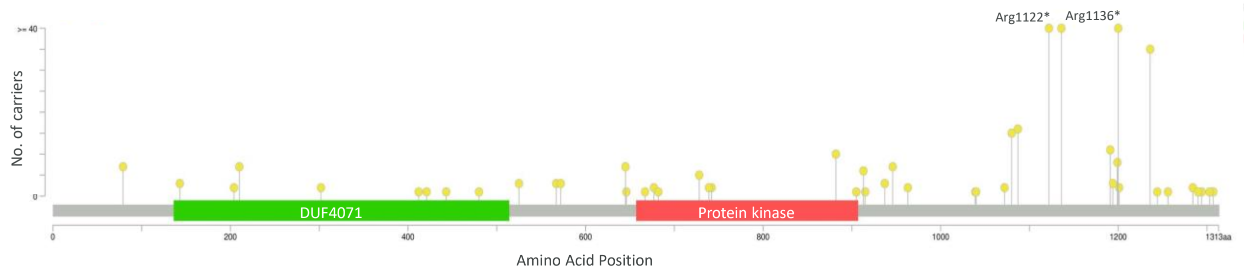

Location of heterozygous PTVs. Lollipop plot of the heterozygous PTVs observed in females of European ancestry in the UK Biobank.

Figure S2

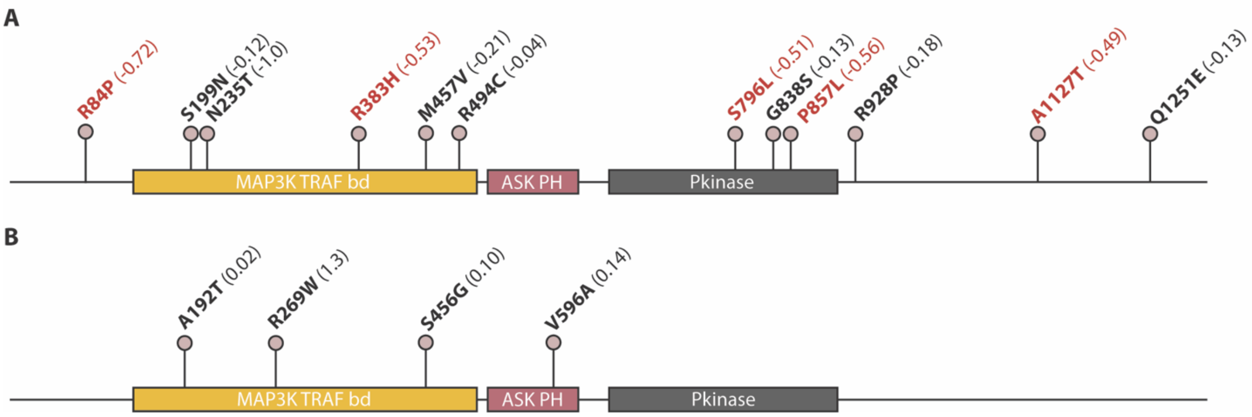

Distribution of missense variant in *MAP3K15*. Missense variants that were nominally associated (P<0.05) with reduced **(A)** and increased **(B)** HbA1C levels in the recessive ExWAS of UKB Europeans. Effect sizes (beta) are included in parentheses. Missense variants colored red conferred effect sizes at least as strong as the Arg1122* hemizygous PTVs (i.e., β≤-0.30).

Figure S3

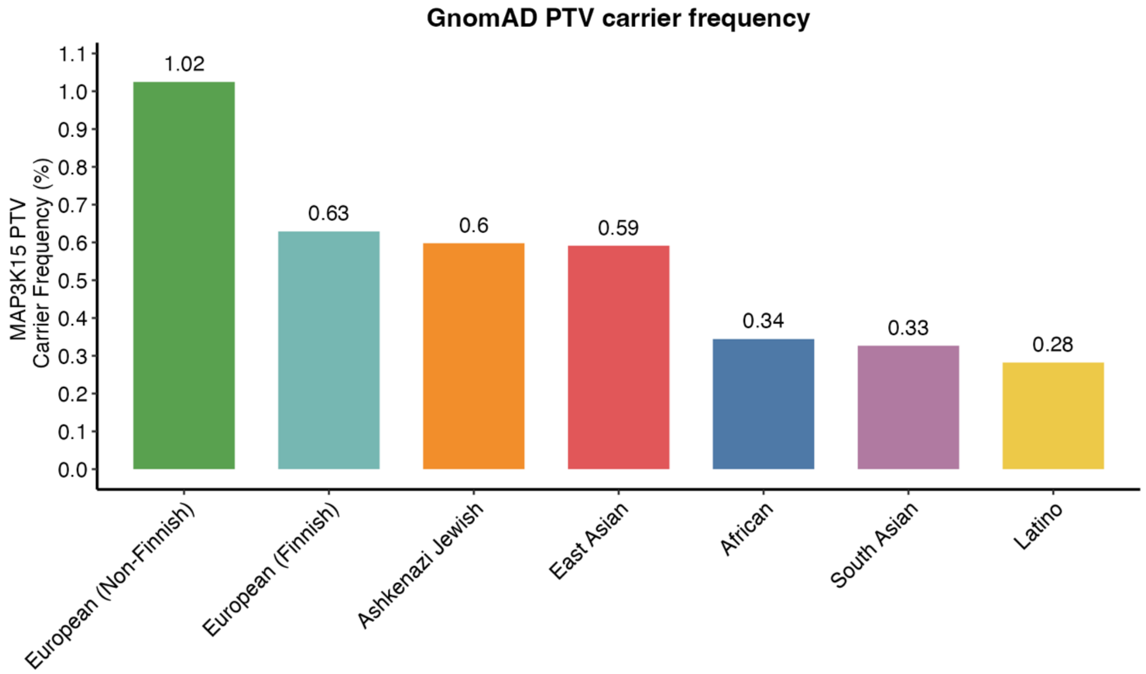

PTV carrier frequencies in gnomAD populations. Bar chart illustrating the different carrier frequencies of protein-truncating variants across the seven global populations included in gnomAD.

Figure S4

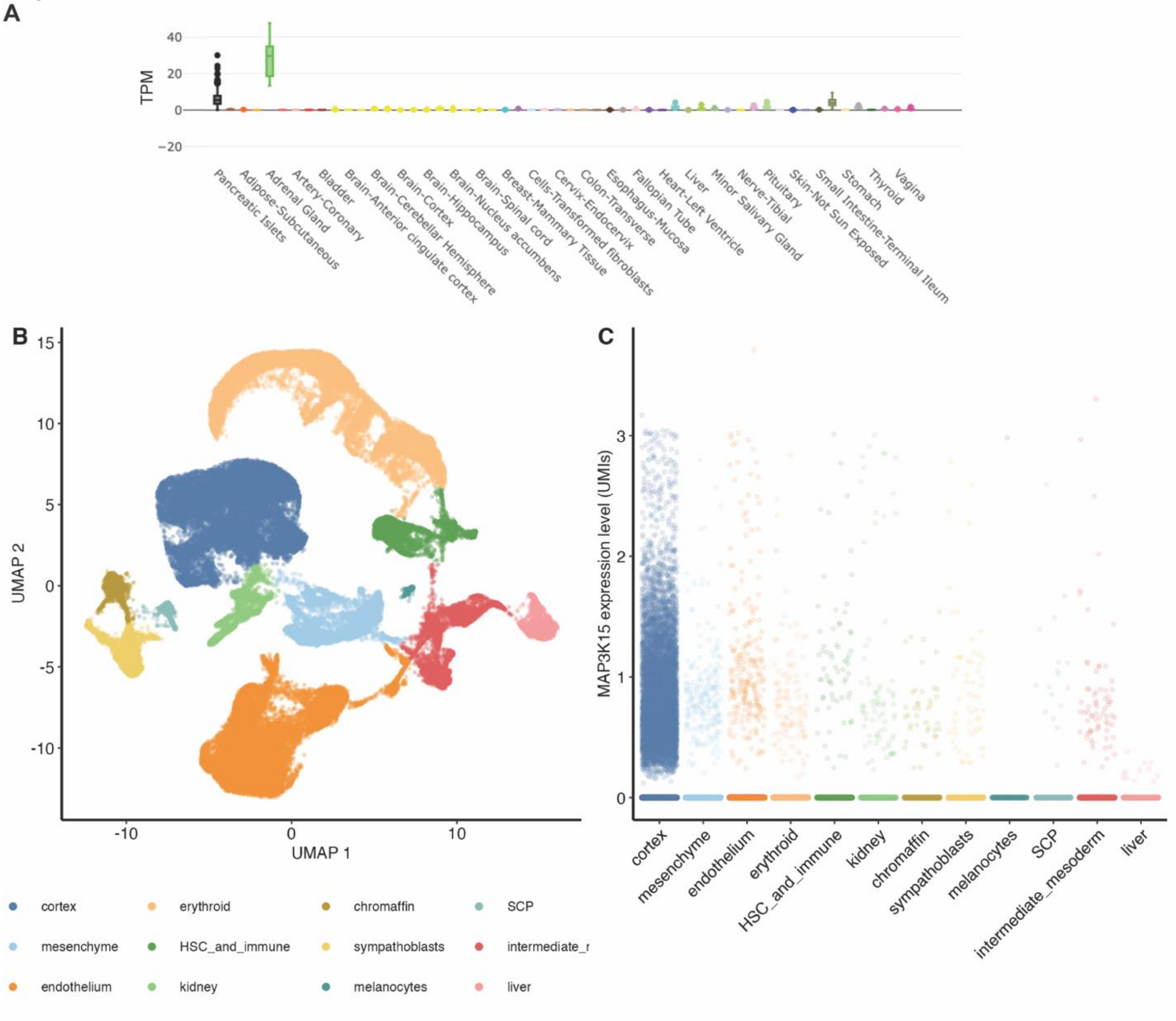

Expression patterns of MAP3K15. (A) Expression of *MAP3K15* in bulk-RNA sequencing data of pancreatic islet cells versus other GTEx tissues. The plot was downloaded from the TIGER database. **(B)** Uniform manifold approximation plot (UMAP) of single-cell RNA-seq data performed on the developing human adrenal cortex, derived from 8 subjects. **(C)** Expression of *MAP3K15* in each major adrenal gland cell type.

Figure S5

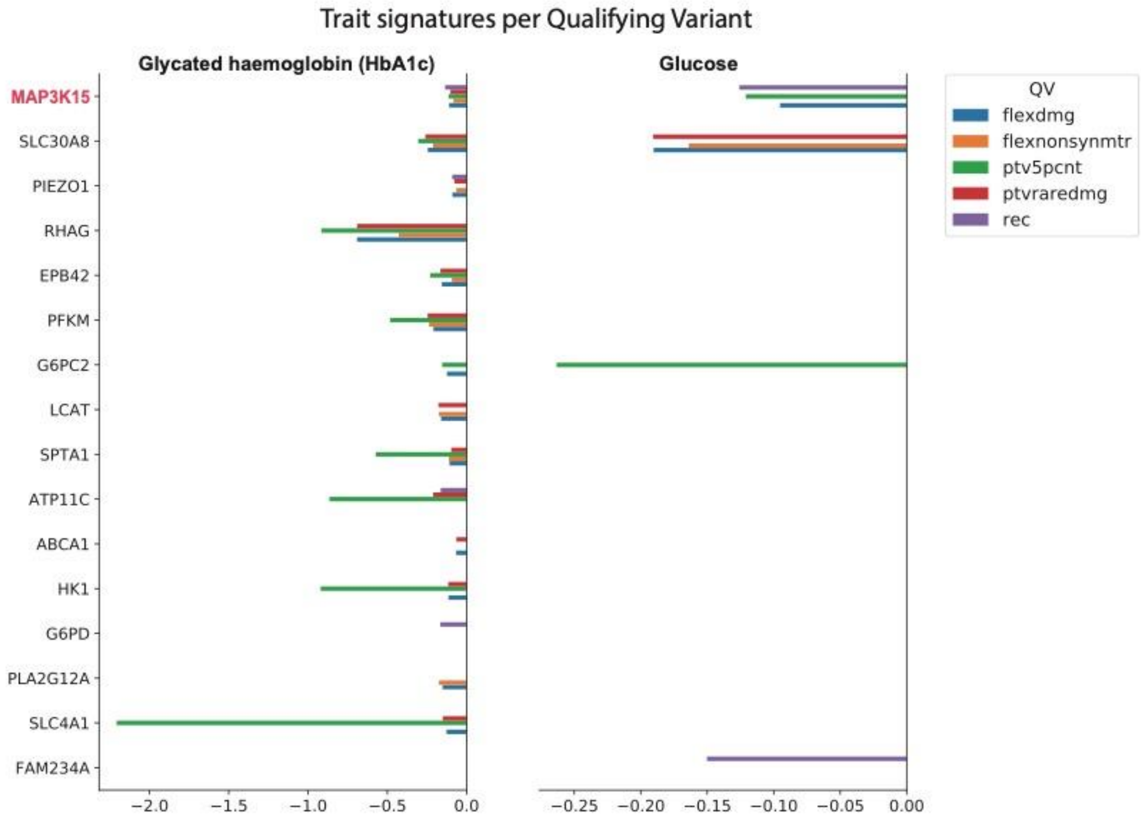

Association signatures with HbA1c and glucose for genes most similar to *MAP3K15*. Comparison of linear regression coefficients for HbA1c and glucose between *MAP3K15* and the genes most similar to it, derived from Gene-SCOUT. All collapsing models for which *MAP3K15* showed significant associations with HbA1c and glucose have been provided.

Table S1
Sample sizes for each binary phenotype in each UKB ancestral group. Provided as an external file.

Table S2
Sample sizes for each quantitative phenotype in each UKB ancestral group. Provided as an external file.

Table S3
Descriptions of the 11 collapsing methods employed in the UKB discovery and pan-ancestry analyses. Provided as an external file.

Table S4
Gene-level collapsing results for associations with the binary diabetes phenotypes in UKB Europeans ($P<0.0001$). P-values were generated via two-tailed Fisher's Exact Test. $P<1x10^{-8}$ is the genome-wide significance threshold (see methods). Provided as an external file.

Table S5
Gene-level collapsing results for associations with the quantitative diabetes phenotypes in UKB Europeans ($P<0.0001$). P-values were generated via linear regression. $P<1x10^{-8}$ is the genome-wide significance threshold (see methods). Provided as an external file.

Table S6
Variant-level ExWAS results for associations with the binary diabetes phenotypes in UKB Europeans ($P<0.0001$). P-values were generated via a two-tailed Fisher's Exact Test. $P<1x10^{-8}$ is the genome-wide significance threshold (see methods). Provided as an external file.

Table S7
Variant-level ExWAS results for associations with the quantitative phenotypes in UKB Europeans ($P<0.0001$). P-values were generated via linear regression. $P<1x10^{-8}$ is the genome-wide significance threshold (see methods). Provided as an external file.

Table S8
All MAP3K15 associations with binary phenotypes for the recessive collapsing model in UKB Europeans. The "T1DM" phenotype refers to a custom-defined phenotype (see methods). P-values were generated via a two-tailed Fisher's Exact Test. $P<1x10^{-8}$ is the genome-wide significance threshold (see methods). Provided as an external file.

Table S9
Associations between *MAP3K15* and binary diabetes phenotypes in hemizygous male PTV carriers and heterozygous female PTV carriers of European ancestry in the UKB. P-values were generated via a two-tailed Fisher's Exact Test. $P<1x10^{-8}$ is the genome-wide significance threshold (see methods). Provided as an external file.

Table S10
Associations between *MAP3K15* and quantitative phenotypes in hemizygous male PTV carriers and heterozygous female PTV carriers of European ancestry in the UKB. P-values were generated via linear regression. $P<1x10^{-8}$ is the genome-wide significance threshold (see methods). Provided as an external file.

Table S11
List of observed *MAP3K15* PTVs in UKB Europeans. Provided as an external file.

Table S12
Conditional analysis results in UKB Europeans. Arg1122* and Arg1136* are the two more common PTVs observed in individuals of European ancestry. Displayed are the associations of each individual variant with diabetes and HbA1C and the association between the remaining *MAP3K15* PTVs when these two more common ones are excluded.

An indel (X-19360844-AAC-A) in the 3'-UTR of *PDHA1*, which overlaps with *MAP3K15*, is also significantly associated with HbA1c. The joint effects of the two MAP3K15 PTVs and the PDHA1 indel were tested (see supplementary note). Provided as an external file.

(OR=Odds Ratio, CI=Confidence Intervals).

Table S13
Individual missense variants that were nominally associated with HbA1C (P<0.05) in the UKB European ExWAS. Also included are association statistics with the self-reported diabetes phenotype for variants included in the binary ExWAS. Provided as an external file.

Table S14
Gene-level collapsing results for binary phenotypes in UKB non-European populations (P<0.0001). AFR = African, SAS = South Asian, EAS = East Asian. P-values were generated via a two-tailed Fisher's Exact Test. $P<1x10^{-8}$ is the genome-wide significance threshold. Provided as an external file.

Table S15
All gene-level collapsing associations with *MAP3K15* under the recessive model UKB non-European populations. AFR = African, SAS = South Asian, EAS = East Asian. Provided as an external file. P-values were generated via a two-tailed Fisher's Exact Test. $P<1x10^{-8}$ is the genome-wide significance threshold. Provided as an external file.

Table S16
Results from the combined, pan-ancestry UKB collapsing analysis for binary diabetes phenotypes. P-values were generated via the Cochran-Mantel Haenszel test. $P<1x10^{-8}$ is the genome-wide significance threshold. Provided as an external file.

Table S17
Results from the combined, pan-ancestry UKB collapsing analysis for quantitative traits. P-values were generated via linear regression (methods). $P<1x10^{-8}$ is the genome-wide significance threshold. Provided as an external file.

Table S18
Results of *MAP3K15* replication analysis for binary phenotypes in the Mexico City Prospective Study (MCPS). Associations were tested using the recessive collapsing model. P-values were generated via Fisher's Exact test. Provided as an external file.

Table S19
Results of *MAP3K15* replication analysis for quantitative phenotypes in the Mexico City Prospective Study (MCPS). P-values were generated via linear regression. Provided as an external file.

Table S20
Association between each common *SLC16A11* variant and self-reported diabetes. Variants were each tested under a separate logistic regression model. Provided as an external file.

Table S21
Association between MAP3K15 and diabetes in carriers and non-carriers of the 17-7041768-G-T *SLC16A11* missense variant. Each association was tested in a logistic regression model. Provided as an external file.

Table S22
Association between MAP3K15 and HbA1C in carriers and non-carriers of the 17-7041768-G-T *SLC16A11* missense variant. Each association was tested in a linear regression model. Provided as an external file.

Table S23
Associations for the Arg1122* PTV in FinnGen. P-values generated via logistic regression. Provided as an external file.

Table S24
Associations between *MAP3K15* and adiposity-related quantitative traits in UKB Europeans in the gene-level recessive collapsing model. Provided as an external file.

Table S25
Associations between *MAP3K15* and NMR Metabolomics in UKB Europeans using the gene-level recessive collapsing model. Provided as an external file.

Table S26
PheWAS results for *MAP3K15* based on the UKB Europeans. Associations were retrieved from the public AstraZeneca PheWAS portal (methods). P-values were generated via Fisher's Exact Test. Provided as external files.

Table S27
List of anti-hypertensive drugs used as covariates in the collapsing analysis.
